# Quality dimensions features for identifying high-quality user replies in text forum threads using classification methods

**DOI:** 10.1371/journal.pone.0215516

**Published:** 2019-05-15

**Authors:** Akram Osman, Naomie Salim, Faisal Saeed

**Affiliations:** 1 School of Computing, Faculty of Engineering, Universiti Teknologi Malaysia, Skudai, Johor, Malaysia; 2 College of Computer Science and Engineering, University of Taibah, Medina, Saudi Arabia; Zapadoceska Univerzita, CZECH REPUBLIC

## Abstract

The Text Forum Threads (TFThs) contain a large amount of Initial-Posts Replies pairs (IPR pairs) which are related to information exchange and discussion amongst the forum users with similar interests. Generally, some user replies in the discussion thread are off-topic and irrelevant. Hence, the content is of different qualities. It is important to identify the quality of the IPR pairs in a discussion thread in order to extract relevant information and helpful replies because a higher frequency of irrelevant replies in the thread could take the discussion in a different direction and the genuine users would lose interest in this discussion thread. In this study, the authors have presented an approach for identifying the high-quality user replies to the Initial-Post and use some quality dimensions features for their extraction. Moreover, crowdsourcing platforms were used for judging the quality of the replies and classified them into high-quality, low-quality or non-quality replies to the Initial-Posts. Then, the high-quality IPR pairs were extracted and identified based on their quality, and they were ranked using three classifiers i.e., Support Vector Machine, Naïve Bayes, and the Decision Trees according to their quality dimensions of relevancy, author activeness, timeliness, ease-of-understanding, politeness, and amount-of-data. In conclusion, the experimental results for the TFThs showed that the proposed approach could improve the extraction of the quality replies and identify the quality features that can be used for the Text Forum Thread Summarization.

## Introduction

**Fig 1:**
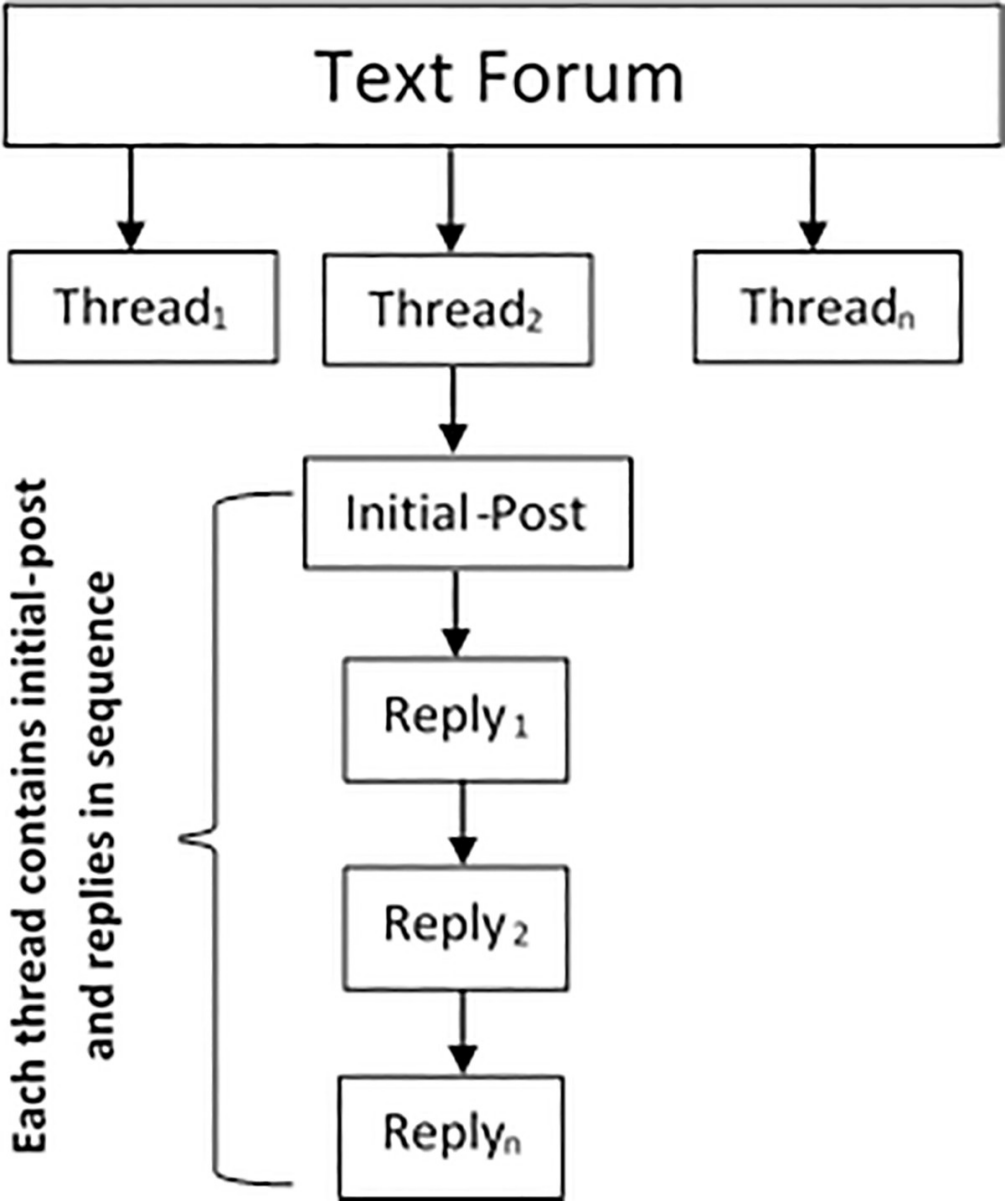
Structure of the text forum threads (Initial-post replies pairs).

An increase in the web services has facilitated the manner in which people accessed and shared the knowledge in the form of User-Generated Content regarding specific subjects on the internet. The Text Forum Threads (TFThs) is the web service wherein the users can initiate discussions by posting Initial-Posts, asking for help and initiating conversations related to specific topics. Other users then read these Initial-Posts and reply accordingly. Hence, the Initial-Post generates many replies in a single thread. The Initial-Post along with its replies are compiled together in one thread. In this study, the authors have referred to the threads as Initial-Posts Replies pairs *(IPR pairs*). Fig 1 describes the manner in which every reply in the thread responds to the Initial-Posts on a particular topic. It can be seen that the discussion thread presented in the forum contains valuable information that is hidden in the forum texts. An effective use of this information in the User-Generated Content is an important topic of research in the field of thread retrieval. Determining important information in the text forums can become very difficult because of the information overloading.

Additionally, the TFThs also have to face the heterogeneity of the content quality. Though the TFThs use monitoring processes for the user contents, it is impossible to monitor millions of text posts [1, 2]. The content from the TFThs is very comprehensive and more objective as compared to other search engines like Google (http://www.google.com) or Yahoo! (http://search.yahoo.com) [3]. One of the main challenges noted in the TFThs is that the quality of the text replies is distributed from irrelevant to relevant (high-quality) replies [4]. This distribution is based on the user requirement and motivation, and diverse user backgrounds and the contents get published without undergoing any prior peer review process [5]. Frequently posted irrelevant replies can affect the user’s impression about the TFThs. Thus, the user has to efficiently navigate through a huge repository of data to find the relevant information [6]. Therefore, an automated assessment of the quality of the content in the TFThs is important. The quality of the content in the TFThs can be improved by distinguishing between the high-quality, low-quality and irrelevant replies. For this purpose, the authors have undertaken this classification task. In general, Quality Dimensions (QDs) are some common features that are applied for enhancing information and the thread retrieval [2, 5, 7–10]. Many QDs features were used in this study for identifying the non-quality (irrelevant), low-quality (parity relevant) and high-quality (relevant) replies in the threads to their Initial-Posts. Additionally, the classification and the feature selection techniques were used for identifying appropriate features for the TFThs, which could help in achieving significant improvements in retrieval performance.

The main objective of this study is to identify the quality features from the variety of quality dimensions that can help in classifying all the user replies to the Initial-Post in the TFThs. Furthermore, a survey on crowdsourcing platform community was conducted to judge the quality of each reply in the thread to Initial-Post.

## 2.0 Background and related work

Identifying the quality features to extract quality replies to an Initial-Post in the TFThs can be difficult. Many studies have been carried out with regards to the issues seen in the TFThs. In this study, the authors have presented a literature review of the studies, which are directly related to this work.

### 2.1 Textual features

One of the important text classification tasks includes the textual feature extraction. The feature extraction is conducted for extracting the important vocabulary words from all the textual data and representing them in an appropriate format that is needed by the machine learning algorithms for further data analysis. Some of the common textual features are the Term Frequency-Inverse Document Frequency (TF-IDF) [11, 12], Bag-of-Words (BoW) [13], word n-grams [14], and the sentiment features [15]. In the past several years, authors have applied several Machine-learning and statistical theory-based techniques for classifying the texts. Machine learning was seen to be a very popular computational method, which was used in many applications for an automated text classification. Some of these applications include the forum thread information extraction [16], post classification [17], thread retrieval [18, 19], thread question-answer pair [20, 21], product reviews [22, 23], text summarisation [24], and forum summarisation [25–27]. Usually, the machine-learning processes are used for determining the features which were helpful for classifying the text conversations [28]. In this study, the authors have investigated the new TFTh classification features known as the Quality Dimensions (QDs) features.

### 2.2 Quality dimensions features

The QDs features refer to the information quality which is vital for the data consumers [29]. Retrieving the quality information in the TFThs is based on the user’s philosophical perspective, like the precise description of the topic, data exclusivity and important content. However, determining the information in the TFThs is complicated due to information overload [10]. The TFThs consist of several thousand posts, which the users find to be time-consuming if they wish to browse or read through the thread. The QDs can measure the quality of each reply in the thread, which can then be used as an importance weighting for identifying the quality replies. It is essential to assess the content quality so that the good-quality content was given a higher weighting value than the low-quality content in the thread summarisation and retrieval systems [30]. Many studies also indicated that controlling the content quality can significantly improve the performance of the functions included in the forums. For example, in [31, 32], the authors stated that using a thread quality process could improve the thread retrieval. Also, in [33], the authors noted that the individuals using a social networking site in the TFThs Community could be a vital factor who improved the search precision regarding the quality replies in any thread. In another study [30], the authors proposed a model for evaluating the usage, reputation, content, temporal and structural features of the user-generated content in the TFThs for identifying the high-quality content. Furthermore, some authors also investigated the structural features along with the author activity features in a TFTh for determining the user knowledge-based adoption decisions (i.e., source credibility and argument quality [32]. In [34], the authors have described four primary user features and used the aggregated post features for classifying the user expertise in all TFThs. In one study [35], the authors used quality features like the number of views and replies, which helped in developing a better forum crawler. Furthermore, in [36], the researchers studied two features which helped in developing the reply graph of the postings and evaluating the relevance of the posting compared to the initial topic. Some authors also aimed to understand the two thread activity features, i.e., decreasing volume of the active participants in the course and the deviation of the discussion, which did not in any way help the faculty members or students [37]. In another study, the authors achieved the quality support in a stack overflowing discussion forum with the help of two features, i.e., the response time and the developer participation [38]. In [39], the authors applied the relevancy dimension and the popularity dimension features for evaluating if the post was related to the topic of discussion or if the post was quoted or answered by other users in the thread. Some studies applied five feature classes, i.e., the lexical, syntactic, surface, forum specific and similarity features for assessing the forum post quality [6, 40]. On the other hand, the appropriateness of the lexical dimension features is not confirmed, since the thread postings of the web forum do not follow correct linguistic rules [39, 40]. One study also determined the linguistic features in various forum communities and noted that they were ineffective when they were investigated using automated quality assessment models since the models could not be adapted for the different writing styles or forum terminologies [5]. In addition to the forum-related applications, some studies stated that quality features were also necessary for retrieving the web documents [41–43]. Many studies indicated that leveraging the quality dimensions can significantly improve the forum summarisation and thread retrieval task [26, 44, 45]. QDs were applied to various text content analytical tasks such as the thread retrieval [18, 19], question-answer pairs in the TFThs [20, 21], and product reviews [22, 23] etc. It must be noted that the QDs could improve the quality of all posts (replies) that were extracted from the discussion threads in the TFThs.

### 2.3 Post retrieval

Several applications are based on the determination of relevant posts in the TFThs, like the post-retrieval in the online forums [17, 46], question-answer pairs [20, 21] and the forum text summarisation [25–27]. These applications are based on the basic concept that for any question or enquiry, there are several potential relevant posts, which differ in their ranking strategy or nature of the enquiry. In [47], the authors were able to extract the quality user replies using the knowledge of Chatbot from the online discussion forums. The authors identified all the replies that were relevant to the thread title using a Support Vector Machine (SVM) classifier and later ranked them with the SVM. In another study [48], the authors described a classification-based process to detect if the Initial-Post in the thread was a question and thereafter all the possible answers to the post were extracted from the replies to the thread. In [49], the author investigated methods for classifying posts based on a predicted quality label. The author used SVMs that were chosen for the classification and regression, because of their diversity, high fault tolerance, and generalizability from other problem domains. In [40], the authors proposed a system for assessing the quality of the forum posts based on the different discussion domains. This system used the SVM classification process which contained features like the lexical, syntactic, surface, forum specific and other similarity features. In [34], the automated categorisation of the online discussion posts was examined using three category sets, i.e., post topic, academic *vs*. general, and seek *vs*. contribute. Though these results were inconclusive, the authors stated that the performance of the process was satisfactory for monitoring the learning progress of the online educational discussion forums. In [50], the authors employed crowdsourcing to judge the quality of online forum threads based on some quality dimensions such as reliability, completeness, and usefulness of the information when people are searching for information. A task similar to the reply classification work carried out in this study was to identify the role played by every individual user message in the online discussion forums by [17]. Here, the authors have aimed to identify the appropriateness of the replies to the inquiries asked in the Initial-Posts in the thread.

### 2.4 Text forum thread summarization

The task of Text Forum Thread Summarization is aimed to provide a brief summary of the whole thread as users can find it difficult to read all the user replies in the thread and retrieve relevant information. The conventional text summarization processes are unable to determine the topic dependencies, scattered topics, drifting of the topics or the text sparseness and are plagued by such problems [51, 52]. In addition, several replies are in the form of short texts, written in an informal language and can contain no punctuations, capitalization, misspellings, grammatical errors, and use many non-standard abbreviations. The informal replies to a post can also be unreliable in comparison to the formal text like the news media. As per the survey conducted in a study, [24], many forum summarization studies considered the thread to a single document, combined all the Initial-Posts with their replies and thereafter the single document summarization approaches were applied to these documents. However, some other studies applied the multi-document summarization approaches after considering the Initial-Posts and their text replies as separate. Furthermore, some other studies applied the thread structural features. For example, in one study, [53], the authors exploited the explicit discourse structure in the discussion threads and thereafter applied the structural discourse relationship between the replies to generate a summary. In another study, [54], the authors observed that the interactions amongst the participants could be grouped as actions involved in seeking help and advice and providing an answer or advice. Rather than summarizing every reply, the summaries must be made for every post related to the post. Statistical methods like dialogue summarization can be very helpful. For example, in one study, [51], the author scored every reply in the discussion thread and selected the most significant replies in the summary. For this purpose, the authors suggested the application of several factors like uniqueness and length of the replies. Furthermore, the ‘term frequency’ was also added as a factor while scoring the replies. In [55], the authors stated that the issues related to the extraction of relevant replies from a discussion thread was a binary classification problem wherein the main task was to classify the replies and determine if they could/ could not be included in the final summary. In study,[56], the authors have established that it would be useful to train an extractive summarization model on a crowdsourced data of a similar model of an expert.

Finally, it can be said that information retrieval in TFThs is a complex issue and discussions related to this issue is not present in the literature [57]. There is a need to present novel quality features of TFThs, which can help in extracting relevant replies (high-quality) and then generating a better text summary.

## 3.0 Methodology

**Fig 2:**
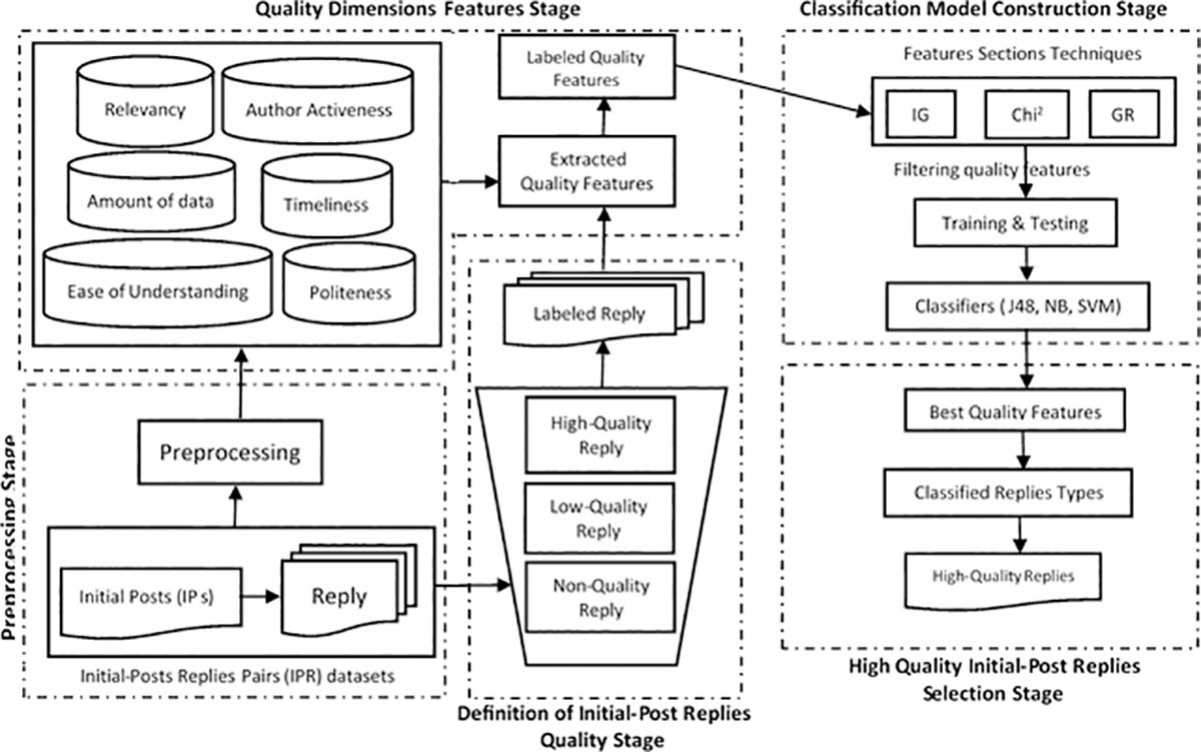
A description of the classified quality initial-post replies model (CQIPRM).

In this study, a Classified Quality Initial-Post Replies Model (CQIPRM) is developed, which consists of five main components, as described in Fig 2. Details of every component is discussed as follows.

### 3.1 Data pre-processing

The initial component of data pre-processing of the threads (IPR pairs) is based on general discussion forums (https://www.tripadvisor.com.my/ShowForum-g28953-i4-New_York.html) and the official discussion forums (http://ubuntuforums.org). Every thread is analyzed with the help of conventional information retrieval processes like stop words removal, tokenization, and stemming, for extracting the terms for the indexing [58]. The raw texts have to be pre-processed for representing them properly and using them effectively in all experiments.

### 3.2 Quality dimensions features extraction

The replies were transformed into their QDs features for every IPR pairs using this component. Section 4 explains the way in which the features were extracted for every reply in the thread. Next, in order to increase the robustness of the overall classification process, the normalization process has been applied for all quality dimensions feature by giving a value between 0 and 1.

### 3.3 Definition of initial-post replies pairs (IPR) quality

In this step, the CrowdFlower Platform (https://www.crowdflower.com/) was used for reading the raw texts and asked to judge the quality of the IPR pairs. The class labels were assigned for every IPR pairs as follows: the replies were labelled as “non-quality reply” if they were irrelevant to the Initial-Posts. They were labelled as “low-quality reply” when they were partially relevant to the Initial-Posts and were called as “high-quality” replies if they were completely relevant to the Initial-Posts. Thereafter, the final class labels for every reply were decided based on the trusted judgement for constructing a precise quality classifier. More details are provided in Section 5.

### 3.4 Classification model construction

Here, the best QDs features for the IPR classification were determined using three classifier techniques. Thereafter, the training and the testing datasets were used in the suggested classification process for constructing the most accurate quality classifier. Further details are presented in Section 7.

### 3.5 High-quality IPR pairs selection

Based on the results of the classification process, the authors selected the IPR pairs, which were categorised as “High-quality” replies.

## 4.0 Description of the quality dimensions features

For understanding and evaluating the quality of the IPR pairs in the TFThs, the replies were classified into three categories. The authors used 28 different quality features, which were divided into six QDs like; relevancy (D1) [33, 36, 39, 40, 59, 60], author activeness (D2) [59–63], timeliness (D3)) [5, 17, 33, 62, 64–66] ease-of-understanding (D4) [13, 21, 65, 67], politeness (D5) [2, 21, 68], and the amount-of-data (D6) [5, 7, 33, 39, 59, 62, 69, 70]. Table 1 summarizes these QDs features, while Table 2 lists the QDs features formulas.

**Table 1:** Quality dimensions features for the TFThs as the classification task.

QDs	Quality Features	Description	Code
D1	The words in the reply overlap with the thread title	Replies with words overlapping with the thread title are more relevant to the Initial-Posts.	F1
	The words in the reply overlap with the Initial-Post.	Replies with words overlapping with the Initial-Posts are more relevant to the Initial-Posts.	F2
	Cosine similarity present between the replies and the thread title.	Replies with words having a higher similarity words with the thread title are more relevant to the Initial-Posts.	F3
	Cosine similarity present between the replies and the Initial-Posts.	Replies with words having a higher similarity words with the Initial-Posts are more relevant to the Initial-Posts.	F4
	Does the reply quote the previous posts?	Replies which quote the Initial-Posts or previous replies are more relevant to the Initial-Posts.	F5
	The centroid of the reply similar to the other replies in the thread.	Replies that have a high similarity score to the thread centroid vector are a better representation of the basic idea behind the Initial-Post.	F6
	The reply overlaps the previous replies.	Replies with words overlapping with the other posts are irrelevant to the Initial-Post.	F7
	Does the reply mention the user-name of the person posting the Initial-Post?	Replies which have mentioned the user-name of the person posting the Initial-Post are considered relevant.	F8
	Does the reply mention the names of other users?	Replies which have mentioned the user-names of other people are considered irrelevant.	F9
	Does the reply have a URL?	Replies with an URL are considered relevant.	F10
D2	Did the user post the Initial-Post?	Replies given by the creator of the Initial-Post are relevant to the Initial-Post.	F11
	Total Number of the Initial-Posts created by the user in the threads.	A high score indicates the user activeness and is relevant to the Initial-Post.	F12
	Total Number of replies given by the user in the current thread.	A high score indicates the user activeness and is relevant to the Initial-Post.	F13
	Total Number of the replies given by the user in all the threads.	A high score indicates the user activeness and is relevant to the Initial-Post.	F14
	Total Number of threads in which the user has participated.	A high score indicates the user activeness and is relevant to the Initial-Post.	F15
	The user’s reputation in all the threads.	This feature determines the mean score of the user replies in all the threads. A high mean score indicates that the user is very important.	F16
D3	Measure the time elapsed between the Initial-Post and the current reply posted.	Age of a reply provides important insights into the relevance of the reply and whether it is still up-to-date. For instance, the replies could become outdated over a period of time, as the discussion in the thread progressed.	F17
	Measure the time elapsed between the posting of the previous and current replies, i.e., Absolute value.	Time elapsed between the posting of the previous and the current replies provides insights about the activeness of the thread. If there is a huge time difference between the replies, then the reply is inferred as inactive, and new replies would be irrelevant to the Initial-Post.	F18
	Measure the time elapsed between the posting of the current and the subsequent replies.	Time elapsed between the posting of the current and the subsequent replies provides insights about the activeness of the thread. A small time difference shows that the current reply has some value since it induces a timely response.	F19
	The position of the text reply in the discussion thread.	Replies at the beginning of the discussion thread define the issue. Also, replies at the end of the discussion thread provide solutions which could be relevant to the Initial-Post.	F20
D4	Does the reply contain the WH question words?	Replies containing the five 5Wh-Q words indicate inquiries or queries and are relevant to the Initial-Posts. The 5Wh-Q words are who, where, what, when, why, and how.	F21
	Does the reply contain a question mark (?)?	Replies containing the question mark (?) indicate queries and are considered to be relevant to the Initial-Posts.	F22
	Does the reply contain an exclamation mark (!)?	Replies containing the exclamation mark (!) reflect ambiguities and are considered relevant to the Initial-Post.	F23
D5	Does the reply contain words of positive feedback? Keywords: Thanks, etc.	Replies containing words of positive feedback reflect the user satisfaction with the earlier replies responding to the Initial-Post and are considered relevant to the Initial-Post.	F24
	Does the reply contain words of negative feedback? Keywords: does not, did not, etc.	Replies containing the negative feedback words reflect the user’s displeasure with the earlier replies responding to the Initial-Post and are considered as irrelevant to the Initial-Post.	F25
D6	Total numbers of words present in the reply	Replies with a high number of words are considered more meaningful and are relevant to the Initial-Post.	F26
	Total numbers of unique words present in the reply.	This feature estimates the amount of data present in the reply by counting the total number of unique words in the reply instead of the total words. High scores for the unique words indicate the relevance of the reply to the Initial-Post.	F27
	Total numbers of sentences present in the reply	This feature estimates the amount of data present in the reply by counting the total number of sentences in the reply. Replies with many sentences clarify the queries in the Initial-Post and are considered very relevant.	F28

**Table 2:** List of QDs features formula used in the TFThs process.

QDs	Code	Formula	Description Symbols
D1	F1	$ReWrdOvlTiThrd=\frac{terms(TiThrd)\cap terms(Rei)}{terms(Rei)}$	*Terms(TiThrd)* = the words in the title of thread. *Terms(Rei*) = the words in reply *i*.
	F2	$ReWrdOvlIPst=\frac{terms(IPst)\cap terms(Rei)}{terms(Rei)}$	*Terms(IPst)* = the words in Initial-Post. *Terms(Rei*) = the words in reply *i*
	F3	$CsinSimilTiThrd=\frac{\sum TiThrd.Rei}{\sqrt{\sum {\left(TiThrd\right)}^{2}}\sqrt{\sum {\left(Rei\right)}^{2}}}$	*Terms(TiThrd)* = the words in the title of thread. *Rei =* reply *i*
	F4	$CsinSimilIpst=\frac{\sum IPst.Rei}{\sqrt{\sum {\left(IPst\right)}^{2}}\sqrt{\sum {\left(Rei\right)}^{2}}}$	*IPst* = Initial-Post. *Rei =* reply *i*
	F5	$ReQuPst=\left\{ \begin{array}{l} 1\;ReQu=termsIpst\\ 0\;otherwise \end{array}\right.$	*ReQu* = words quoted by the reply *termsPst* = Posts words
	F6	$CentrReToRei=\sum_{i=1}^{n}\frac{i}{n}{Re}_{i}$	*i =* the order of replies, *Rei =* reply *i*
	F7	$OvlRePrRe=\frac{terms(Rei)\cap terms(PrRei)}{terms(PrRei)}$	*Terms(PrRei*) = the words in previous replies. *Terms(Rei*) = the words in reply *i*.
	F8	$ReMeIPst=\left\{ \begin{array}{l} 1\;Rei=IPstCrtr\\ 0\;otherwise \end{array}\right.$	*Rei =* reply *i* *IPstCrtr* = name of the Initial-Post creator
	F9	$ReMeRei=\left\{ \begin{array}{l} -1\;RejMe=ReiCrtr\\ \;0\;otherwise \end{array}\right.$	*RejMe = current reply j* *ReiCrtr* = name of creator reply *i*.
	F10	$ReUrl=\left\{ \begin{array}{l} 1\;URL\in Rei\\ 0\;otherwise \end{array}\right.$	*URL =* Website link in the reply
D2	F11	$UsrIpst=\left\{ \begin{array}{l} UrRei=iPstCrtr\;1\\ UrRei<>iPstCrtr\;0 \end{array}\right.$	*UrRei =* reply i creates by user. *iPstCrtr =* name of users created Initial-Posts.
	F12	$NumIPstByUsr=\left\{ \begin{array}{l} UrRei=iPstCrtr\;1\;iPst=iPst+1\\ UrRei<>iPstCrtr\;0\;continue \end{array}\right.$	*UrRei =* reply i creates by user. *iPstCrtr =* name of users created Initial-Posts.
	F13	$NumReiByUsrThrd=\left\{ \begin{array}{l} UrRei=UrCrtr\;1\;Rei=Rei+1\\ UrRei<>UrCrt\;0\;continue \end{array}\right.$	*UrRei =* reply creates by user. *UrCrtr =* name of users in the thread.
	F14	$SumAllRplyByUsr=\sum_{T=1}^{M}\sum_{R=1}^{N}NoRei$	*R =* replies, *T =* threads *NoRei* = number of replies created by user
	F15	$NoTrdPrtspt=\sum_{T=1}^{M}\sum_{R=1}^{N}ThrdPrt$	*R =* replies, *T =* threads *ThrdPrti* = the number of threads participated by each user.
	F16	$AR=\frac{\sum_{Ur=1}^{n}UsrRply-\sum_{IP=1}^{m}UsrIP}{\sum_{r=1}^{k}AllRply}$	*UsrRply* = number of replies by user in the threads. *UsrIP* = number of Initial-Posts by user in the threads. *AllRly* = number of all replies in the threads.
D3	F17	*TimToIpst = ReiTim–IPstTim*	*ReiTim =* Reply Date *IPstTim =* Initial-Post date
	F18	$TimToPrevPst=\left\{ \begin{array}{l} ReiTim-PreReiTim\;PreRei\neq null\\ 0\;otherwise \end{array}\right.$	*ReiTim =* Reply Date *PreReiTim* = previous reply date
	F19	$TimToPrevPst=\left\{ \begin{array}{l} ReiTim-NxtReiTim\;NxtRei\neq null\\ 0\;otherwise \end{array}\right.$	*ReiTim =* Reply Date *NxtRei* = Next reply date
	F20	$RePosition=\frac{{Re}_{i}}{\sum_{i=1}^{n}NoRei}$	*Rei =* Position of reply. *NoRei* = Number of replies in the thread.
D4	F21	$ReWhQu=\left\{ \begin{array}{c} 1\;WhQ\in Rei\\ 0\;otherwise \end{array}\right.$	*Rei =* reply *i* *WhQ = 5WH-Q words (*what, where, when, why, who, how*)*
	F22	$ReQuMarks=\left\{ \begin{array}{l} 1\;QuMarks\in Rei\\ 0\;otherwise \end{array}\right.$	*Rei =* reply i *QuMarks =* question mark (?)
	F23	$ReExMarks=\left\{ \begin{array}{l} 1\;ExMarks\in Rei\\ 0\;otherwise \end{array}\right.$	*Rei =* reply i *ExMarks =* exclamation marks (!)
D5	F24	$RePosWrd=\left\{ \begin{array}{l} 1\;PosWrdRei\in Rei\\ 0\;otherwise \end{array}\right.$	*Rei =* reply i *PosWrdRei =* positive feedback words
	F25	$ReNegWrd=\left\{ \begin{array}{l} 1\;NegWrdRei\in Rei\\ 0\;otherwise \end{array}\right.$	*Rei =* reply i *NegWrdRei* = negative feedback words
D6	F26	$NoWrdRe=\sum_{w=1}^{n}WrdTxt$	*w* = order of words in a reply *WrdTxt* = Number of words in a reply
	F27	$NoUnWrdRe=\sum_{uw=1}^{n}UnWrdTxt$	*uw* = order of unique words in a reply *UnWrdTxt* = Number of unique words in a reply
	F28	$NoSenRe=\sum_{se=1}^{n}SentTxt$	*se* = order of sentences in a reply *SentTxt* = Number of sentences in a reply.

The main motivation for using the QDs features for the IPR pairs was described in Table 1, in the following sub-sections.

### 4.1 Relevancy dimension (D1)

This is a very important dimension, which builds the user perception about the relevancy of the reply given to an Initial-Post. The relevance of the reply reflects its suitability to the post. High-quality replies must have similarities and contain overlapping words to the Initial-Post. However, the irrelevant and off-topic replies show a low similarity score. Moreover, if the reply quotes the earlier replies or the username of the person posting the Initial-Post, it indicates that the reply is in direct response to the problems mentioned in the Initial-Post. In addition, replies with an URL are considered very relevant. Table 1 summarizes all quality features addressed by the relevancy dimension.

### 4.2 Author activeness dimension (D2)

The contributions of many authors (participants) in the thread generate a significant source of new ideas, which can improve the quality of the content for all the user replies [49]. The D2 features focus more on assessing the activities and the contribution of the authors in responding to the Initial-Post. An active author has a wide experience. When the author interacts with the person, who has posted the Initial-Post that would increase the trust between them. However, in the case the author interacting with other authors in the discussion thread, it has a negative effect because it often leads to deviation from the original topic in the Initial-Post and provokes a new discussion or a new topic unrelated to the inquiry (issue) raised in the Initial-Post. The activities of the authors indicate their involvement and commitment to the issue raised in the Initial-Post of each thread. Furthermore, the credibility of the authors is measured by assessing the amount of personal information provided by them. The authors are accountable to their replies and content that they created. Table 1 presents the features addressed by the author activeness dimension.

### 4.3 Timeliness dimension (D3)

The D3 dimension quantifies the timeliness of the reply by assessing the reply age. The time between the IPR pairs indicates if the replies are still relevant and up-to-date. Furthermore, the temporal feature of the thread reflects the relevancy of the user replies to the Initial-Post based on its age. The timeliness is measured by the reply position in the thread compared to other user replies. These features determine the visibility of any reply (whether it is displayed on the first page of the discussion thread). Table 1 summarizes the features included in the timeliness dimension.

### 4.4 Ease-of-understanding dimension (D4)

The D4 dimension evaluates if the contents of the reply to the Initial-Post can be understood easily. The features addressed by this dimension help in determining the types of the question and answer the replies [39]. A higher number of replies containing the Wh-Qs or question marks show that the issue raised in the Initial-Post needs more clarification from the Initial-Post creator. This also indicates that the replier needs further details if the issue raised in the Initial-Post needs to be resolved completely. For example, *“Do you want to stay in any particular part of Rochester*? *Downtown*? *Suburbs*?” (https://www.tripadvisor.com.my/ShowTopic-g48503-i903-k1986305-Best_area_to_stay_for_nightlife-Rochester_Finger_Lakes_New_York.html). On receiving additional details, any user who has knowledge could provide a possible answer or valid response to this issue raised in the Initial-Post. It might suggest a solution and share his experience in the next reply. Hence, replies consisting of any one of the 5Wh-Qs are seen to be relevant and important for the Initial Post. All the features assessed by the ease-of-understanding dimension have been presented in Table 1.

### 4.5 Politeness dimension (D5)

The D5 dimension measures the user politeness while expressing opinions, responding, and while addressing other replies. This feature helps in determining the words of appreciation or denial used in the user replies. For example, replies with words like "This worked!" or "Thank you" reflect the user politeness as they are appreciative. This would also help others to determine if the replies were relevant. Table 1 summarizes the features used by the politeness dimension.

### 4.6 Amount-of-data dimension (D6)

The D6 dimension estimates the quantity of the information provided in the user replies. It measures the word count in the replies as it shows a degree of the user participation. It is also seen that replies with high word count provide a sufficient amount of knowledge and contain productive discussion matter [71], and were relevant to the Initial-Post. Table 1 presents the features addressed by the amount-of-data dimension.

## 5.0 Human judgment

A classification of the user replies based on their response to the Initial-Posts could be helpful in the TFThs. In this study, the researchers have described the manner in which the reply classification information is used in the TFThs system. They have incorporated the class label information about the replies in the dataset for determining if it improved the TFThs system. Based on the human judgments, the replies are classified into three classes, i.e., to evaluate each reply in the thread, i.e., non-quality, low-quality, and high-quality replies. In Table 3, the authors have presented an example of the discussion thread containing an Initial-Post and replies with the class Labels, which were represented by the nominal values. The class labels display the information below:

- The High-quality Replies were completely relevant and provide a good response to the Initial-Post. They were trustworthy, informative and fact-based.
- The Low-quality Replies were partially relevant and provided satisfactory responses to the Initial-Post. The replies were sensible and provided some information.
- The Non-quality Replies were completely irrelevant, subjective and uninformative and provided no useful knowledge in response to the Initial-Post.

In Table 3 is mentioned below, Rows 1 and 2 consist of the title and Initial-Post and provide a context for the discussion thread. They were not a target class. The Table consists of a discussion thread (https://www.tripadvisor.com.my/ShowTopic-g60763-i5-k3128263-How_to_get_from_JFK_to_New_Rochelle-New_York_City_New_York.html) where every reply is labelled with a proper class label. The three classes were used for judging the quality of the user replies in the TFThs. The authors studied 100 threads, including the user replies for the 100 Initial-Posts along with their respective class labels.

**Table 3:** An Example of the discussion thread containing class labels for the IPR pairs.

Topic Title: *How to get from JFK to New Rochelle*	
**Initial-Post:** *Can someone please advise the best way to get from JFK to New Rochelle using public transport*? *I know that by Taxi it is $80–85 USD which is quite expensive*.	
**High-Quality Reply**	**Reply1:** *Your combined cost of getting from JFK to New Rochelle and back to JFK—using the Airport Express Service bus and Metro North—will be $40–45 round-trip*, *depending on whether you'll riding the Metro North train at times it deems to be peak or off-peak*. *The New Rochelle train station is only 1/2 mile from the Residence Inn*, *and according to Metro North’s page re*: *the New Rochelle station*, *Bluebird Taxi has its office right at the train station*. *Sounds easy peasy* :*-)*
**Low-Quality Reply**	**Reply2:** *What are you going to do when you get to New Rochelle*? *Since it is not in the city you will need transportation of some sort to get around*. *In that case you may want to rent a car at JFK and drive*. *The one day cost would be equal to the taxi fare and you will be in control*.
**Non-Quality Reply**	**Reply3:** *One more thing*, *would I have to be going up and down a lot of stairs*? *I was thinking about my luggage* …

## 6.0 Experiments design

In this study, two datasets were used—the online TripAdvisor forum (https://www.tripadvisor.com.my/ShowForum-g28953-i4-New_York.html) for New York City (NYC) along with the online Ubuntu Linux distribution forum (http://ubuntuforums.org) [17]. The two datasets comprised of discussion threads, where every IPR pairs generated a thread. The statistics for both the datasets have been provided in Table 4 by [17].

**Table 4:** Statistics for the trip advisor forum and the ubuntu linux distribution forum.

	NYC	Ubuntu
**Number of threads**	83072	113277
**Number of users**	39454	103280
**Number of replies**	590021	590021

The authors randomly chose 100 discussion threads from the NYC and the Ubuntu datasets, with 816 and 773 replies, respectively. As the judgment quality for the IPR pairs was unavailable, the authors conducted a survey on crowdsourcing platform community (https://www.crowdflower.com/) to judge the quality of each reply in the thread to initial post. This platform was used for assigning class labels to each posted reply, as mentioned in Section 5.

In the case of the NYC dataset, the authors noted that 342 (42%) replies were of a high-quality, 303 (37%) replies were low-quality and 171 (21%) replies were non-quality. In the case of the Ubuntu dataset, 444 (57%) replies out of 773 replies were high-quality, 233 (30%) replies were low-quality while 96 (13%) replies were non-quality. Table 5 summarizes the distribution of the user replies in various classes for the two datasets.

**Table 5:** A distribution of the user replies in various classes for the two datasets.

Reply Class	NYC		Ubuntu	
Non-Quality	171	21%	96	13%
Low-Quality	303	37%	233	30%
High-Quality	342	42%	444	57%
Total	816	100%	773	100%

### 6.1 Classification algorithm

Due to the availability of texts in digital form and the increasing need to access them properly, the text classification was seen to be an important task [72], to find interesting information on the internet. It is very complicated and time-consuming to develop the text classifiers manually, hence, classifiers can be studied using samples [73, 74]. In order to classify the IPR pairs quality, the authors used many supervised machine learning algorithms [74] like the Support Vector Machine (SVM) [75], the Naïve Bayes (NB) [72] and the Decision Tree (J48) [76].

#### 6.1.1 Support vector machines (SVM)

There are more than 10,000 features that need to be looked at when learning about text classifiers. As support vector machines are not really dependent on the number of features, they are considered optimum to handle such large feature spaces [77]. Thus, SVM is regarded to be helpful in text classification. The SVM is considered to be an effective supervised classification algorithm. SVMs employs the idea of drawing a line called as hyperplane which segments a dataset into classes in the best way. Support vectors can be defined as the data points closest to the hyperplane. If these points pertaining to a dataset be removed, it would also change the dividing hyperplane’s position. Therefore, they are referred to as the critical elements pertaining to a dataset. Intuitively, the greater the distance of these data points from the hyperplane, the better the chance they have been properly classified. Thus, when addition of new testing data is done, whichever side of the hyperplane it lands, it would define the assigned class subsequently. The margin can be defined as the distance between the hyperplane and the closest data point from either set. The key aim here is to select a hyperplane that has the highest possible margin between the hyperplane and any point inside the training set, which provides a higher chance of new data to be classified suitably. Inside the margin, there are no data points at any time. The SVM was initially developed for solving 2-class problems. However, many techniques were later developed that extended the SVM to the multi-class datasets.

#### 6.1.2 Naïve bayes (NB)

The NB classifier is a simple probabilistic classifier based on a common assumption that all features are independent of each other, given the class variable [78]. NB classifier performs well especially for problems that are linearly separable and fairly well for problems that are nonlinearly separable[79]. It is suitable for text classification as well as achieving good performance when dealing with high-dimensional feature spaces.[77, 80]. NB has the feature to learn the pattern of assessing a set of documents that have been already classified. Then, the contents are compared with the list of features that allows assigning the documents to their correct class [81]. The NB classifier is based on theorem by Bayes and the theorem of total probability [82]. For example, consider a probability in which a document d is represented by the vector X, X = {x_1_, x_2_,…x_n_}, wherein n refers to the number of features. The probability of the sample belonging to a specific class is calculated using the following formula:

$$P\left(c|x\right)=\frac{P\left(x|c\right)P\left(c\right)}{P\left(x\right)}$$ (1)

$$P(c|x)=P(x_{1}|c)\times P(x_{2}|c)\times \ldots \ldots \times P(x_{n}|c)\times P(c)$$ (2)

Wherein, P(c|x) represents the probability of class, c; for a feature, x. Let P(c) be the probability of the class, while P(x) is the feature probability. P(x|c) refers to the probability that the feature belongs to a specific class.

#### 6.1.3 Decision trees (J48)

J48 algorithm is based on decision tree. The decision trees learning approach uses the structure of trees for classifying the samples. Decision trees starts with one node, which then branches into other possible results. The leaf node also refers to class labels. On the other hand, a branch indicates the feature conjunctions, while the nodes (or non-leaf nodes) represent the conditional tests on a feature.

The classification results for three algorithms were obtained with the help of the 10-fold cross validation that is used for data mining. The classifier performance was studied based on the Precision (P), Recall (R) and the F-1 measure. The authors pre-processed the IPR pairs after removing the HTML tags and then stemming the words with Porter’s stemmer [83]. Normal stop words in the English Language were used. For determining the best QDs features for classifying the replies to the Initial-Post, the feature selection methods (filter methods) are commonly used in text classification to reduce the numbers of text features and improve the efficiency and accuracy of classifiers[84]. More details are presented in the next Section.

### 6.2 Quality feature reduction

In a text classification process, the main issue that arises includes the feature space high dimensionality. Generally, a text domain consists of numerous features. These texts can be represented as the vector containing *m* elements, wherein *m* refers to the feature number, which are usually text words. A majority of the features are irrelevant and not helpful for the task classification process [72]. A few of the features can significantly decrease the classification accuracy. Also, high feature number can slow the classification process, or make a few classifiers inapplicable. Here, the authors investigated the above-mentioned quality dimensions features. For decreasing the number of features and removing irrelevant features, the filters define a feature subset. This subset is vital for eliminating the noisy, irrelevant, non-valuable and redundant features and also helps in- (1) Improving the classification accuracy and runtime [85]; (2) Reducing the feature space size and improving the quality of the classification method [86].

In this study, the authors have applied the features selection techniques (filter methods) based on a statistical measure like the Information gain [87], Chi-square [88] and the Gain Ratio [89] for selecting the important features. These methods assign a score to every feature and rank all features, wherein all high-ranked features are chosen and applied to the classifier [84]. The three filter methods are described below.

#### 6.2.1 Information gain (IG)

This method determines the decrease in the entropy by taking into account the presence or the absence of a specific feature in every user's reply in a thread. IG is used for selecting the test features in every class. IG aims to—(1) Select the features with several values; (2) Decide the feature order; (3) Identify the features in a specific set of training features which helps in TFThs classification.

Furthermore, the IG estimates the gain between the *i*^*th*^ feature *f*_*i*_ and the class label, C, with the help of the Eq (3) as follows:

$$IG(f_{i},c)=H(f_{i})-H(f_{i}|C)$$ (3)

Wherein H (*f*_*i*_) refers to the entropy of *f*_*i*_; while H(*f*_*i*_ | C) refers to the entropy of *fi* after determining the C value. Hence,

$$H(f_{i})=-\sum p(f_{i})\log_{2}\left(p(f_{i})\right)$$ (4)

Eq (4) describes the uncertainty in the feature set which is selected. For a class label that is observed, the conditional entropy is determined as:

$$H(f_{i}|C)=-\sum_{j}p(f_{i}|c_{k})\log_{2}\left(p(f_{i}|c_{k})\right)$$ (5)

This indicates that after observing C, class label, the uncertainty is decreased in the features that have to be selected.

#### 6.2.2 Chi-square (Chi^2^)

This method measures the absence of independence between the *f*_*i*_ feature in Class c_k_. It is used as the (i) goodness-of-fit test which is used for a set of data and a specific statistical distribution, or (ii) A test of independence or relation between 2 variables or factors [90]. For solving the feature selection issue in the text classification, the Chi-Square value helps in ranking the features based on their use and cannot determine the statistical dependence of the *f*_*i*_ feature and c_k_ class [90]. By considering a 2-way contingency table for the *f*_*i*_ feature and c_k_ class, wherein A denotes the number of times the *f*_*i*_ and c_k_ co-occurred, while B denotes the number of times the *f*_*i*_ occurred without the c_k_; C refers to the number of times the c occurred without *f*_*i*_; D refers to the number of times neither the c_k_ nor the *f*_*i*_ occurs, and N refers to the total number of thread replies. Thus, Chi-square value is determined as:

$$x^{2}(f_{i},c_{k})=\frac{N\times {\left(AD-CB\right)}^{2}}{\left(A+C)\times (B+D)\times (A+B)\times (C+D\right)}$$ (6)

#### 6.2.3 Gain ratio (GR)

The GR is seen to improve the IG measure as it can offer a normalised score of the contribution of the feature to the optimal IG-based classification decisions. The GR is used as an iterative procedure, wherein small feature sets are selected in an incremental manner. These iterations are terminated when only a predetermined number of features are left. The GR is used as a disparity measure, and a high GR ratio indicates that the feature is useful for the classification process. Thus, GR can be computed as:

$$GR\;(a)=\frac{Information\;gain\;(a)}{SplitInfo\;(a)}=\frac{IG\;(a)}{K\;(a)}$$ (7)

$$K(a)=-\sum_{i=1}^{v}\frac{\left|P_{i}\right|}{\left|P\right|}{.\log}_{2}\frac{\left|P_{i}\right|}{\left|P\right|}$$ (8)

Where, K (a) can be calculated by splitting the training examples into *v* partitions, wherein *v* refers to the outcome of the test applied on the feature *a*; while | Pi | refers to the number of replies present in the tanning dataset, *P*.

## 7.0 Results and discussion

In the subsequent subsections, descriptions regarding the classification result, reduction result and more discussions on the result via confusion matrix have been provided. Moreover, this work has been compared by the authors with a related work (baseline).

### 7.1 Classification result

The results for the classification of the IPR pairs for the NYC and the Ubuntu datasets with the help of the SVM, NB and the J48 classifiers are presented in this section. The effects of the QDs features on the classification of the IPR pairs were investigated. All the classification experiments were conducted using a single QD features at a time as well as aggregating all QDs features.

**Fig 3:**
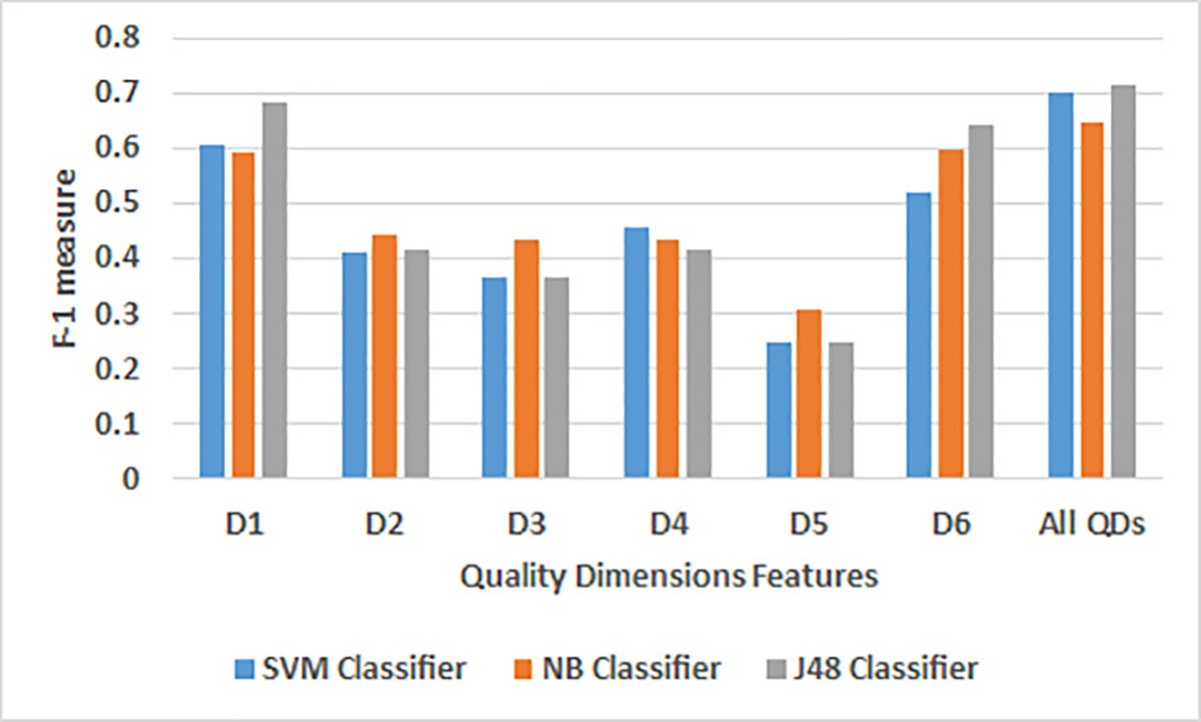
A comparative analysis of the individual QD features using the three classifiers for the NYC.

The classification results for the NYC dataset for every QD features are presented in Table 6 and Fig 3. According to the results, the three classifiers (SVM, NB, and J48) showed the best individual performance for the relevancy dimension features (D1) and the best among the three classifiers is J48 classifier based on its Precision (0.688), Recall (0.692) and the F-1 measure (0.684). The second best individual performance was achieved by the three classifiers as well for the amount-of-data dimension features (D6) and also the best classifier for this dimension is the J48 based on its Precision (0.647), Recall (0.651) and the F-1 measure (0.645). Furthermore, all the QDs features showed a better performance for the precision, recall and F-1 measure compared to the individual QD features using the SVM, NB and the J48 classifiers. The J48 classifier was seen to be the best classifier and showed high values for the precision (0.730), recall (0.723) and F-1 measure (0.716) for all the QDs features.

**Table 6:** Results for the SVM, NB and the J48 Classifiers for the NYC dataset.

QDs	SVM Classifier			NB Classifier			J48 Classifier		
	Avg-P	Avg-R	Avg-F	Avg-P	Avg-R	Avg-F	Avg-P	Avg-R	Avg-F
**D1**	0.618	0.62	0.606	0.642	0.625	0.596	**0.688**	**0.692**	**0.684**
**D2**	0.473	0.48	0.411	0.464	0.487	0.445	0.51	0.489	0.418
**D3**	0.333	0.433	0.367	0.443	0.466	0.434	0.407	0.438	0.366
**D4**	0.485	0.479	0.456	0.443	0.466	0.434	0.375	0.469	0.416
**D5**	0.176	0.419	0.248	0.249	0.403	0.307	0.176	0.419	0.248
**D6**	0.489	0.583	0.521	0.623	0.605	0.597	0.647	0.651	0.645
**All QDs**	0.709	0.706	0.704	0.666	0.656	0.651	**0.730**	**0.723**	**0.716**

**Fig 4:**
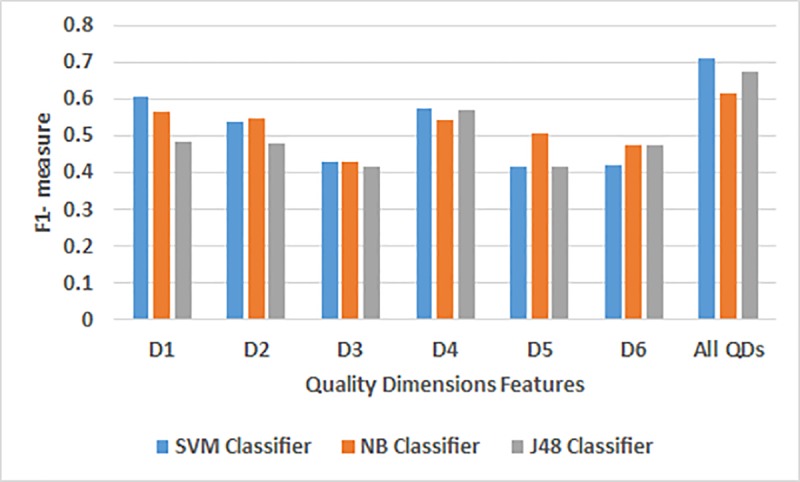
A comparative analysis of the individual QD features using the three classifiers for the Ubuntu dataset.

Table 7 and Fig 4 show the classification results for the Ubuntu dataset for all the QD features. According to the results, the three classifiers achieved high performance for specific QDs features. SVM classifier showed the best individual performance for the relevancy dimension features (D1) based on its Precision (0.681), Recall (0.661) and the F-1 measure (0.606) compared to the other QDs for the same classifier. Comparatively, the SVM classifier is also the best among the three classifiers. Likewise, NB classifier showed the best individual performance for the relevancy dimension features (D1) based on its F-1 measure (0.568) compared to the other QDs for the same classifier. However, for the ease-of-understanding dimension features (D4), J48 classifier showed the best individual performance based on its Precision (0.535), Recall (0.614) and the F-1 measure (0.572) compared to the others QDs for the same classifier. Therefore, we observed there are no specific type of QDs features that achieve high performance across three classifiers based on its precision, recall and the F-1 measure. Furthermore, all the QDs features showed a better value for the precision, recall and the F-1 measure as compared to the individual QD features for all the classifiers. The SVM classifier showed the best values for the precision (0.754), recall (0.735) and the F-1 measure (0.712) for all the QDs features.

**Table 7:** Results for the SVM, NB and the J48 Classifiers for the Ubuntu dataset.

QDs	SVM Classifier			NB Classifier			J48 Classifier		
	Avg-P	Avg-R	Avg-F	Avg-P	Avg-R	Avg-F	Avg-P	Avg-R	Avg-F
**D1**	**0.681**	**0.661**	**0.606**	0.586	0.585	0.568	0.515	0.576	0.487
**D2**	0.575	0.605	0.541	0.539	0.578	0.547	0.468	0.577	0.482
**D3**	0.572	0.578	0.43	0.682	0.578	0.43	0.33	0.574	0.419
**D4**	0.539	0.617	0.575	0.514	0.598	0.543	0.535	0.614	0.572
**D5**	0.33	0.574	0.419	0.473	0.567	0.507	0.33	0.574	0.419
**D6**	0.632	0.576	0.422	0.697	0.583	0.478	0.403	0.587	0.477
**All QDs**	**0.754**	**0.735**	**0.712**	0.626	0.642	0.617	0.689	0.702	0.677

### 7.2 Reduction result

The result of the significance of every individual quality feature for the classification of the IPR pairs was also studied. Every quality feature was studied individually after calculating the IG, Chi^2^ and GR values for the class labels, and ranking the quality features based on their IG, Chi^2^ and GR values. The top 12 quality features used for classifying the IPR pairs for the NYC and the Ubuntu datasets is listed in Table 8, the lists do not include the Timeliness dimension features (D3) for the two datasets that were studied. Also, the lists do not include the Politeness dimension features (D5) for the NYC dataset. This means that these quality dimensions features are not important for the classification of the IPR pairs to identify relevant (quality) replies. Theses QDs achieved low scores and ranked at the bottom of the list wherein the lowly ranked features are irrelevant and unnecessary for these domains.

**Table 8:** Top 12 quality features for the NYC and Ubuntu datasets that were ranked based on their IG, Chi2 and GR values.

	NYC Dataset			Ubuntu Dataset		
	IG	Chi2	GR	IG	Chi2	GR
1	F27	F27	F27	F22	F27	F13
2	F28	F28	F28	F27	F22	F27
3	F4	F4	F4	F11	F4	F22
4	F1	F1	F11	F4	F11	F4
5	F11	F11	F1	F1	F1	F11
6	F2	F2	F13	F12	F12	F1
7	F9	F9	F9	F21	F21	F10
8	F13	F13	F2	F28	F28	F12
9	F3	F12	F12	F13	F13	F21
10	F12	F3	F3	F10	F25	F28
11	F21	F21	F21	F24	F24	F24
12	F15	F15	F15	F25	F15	F25

**Fig 5:**
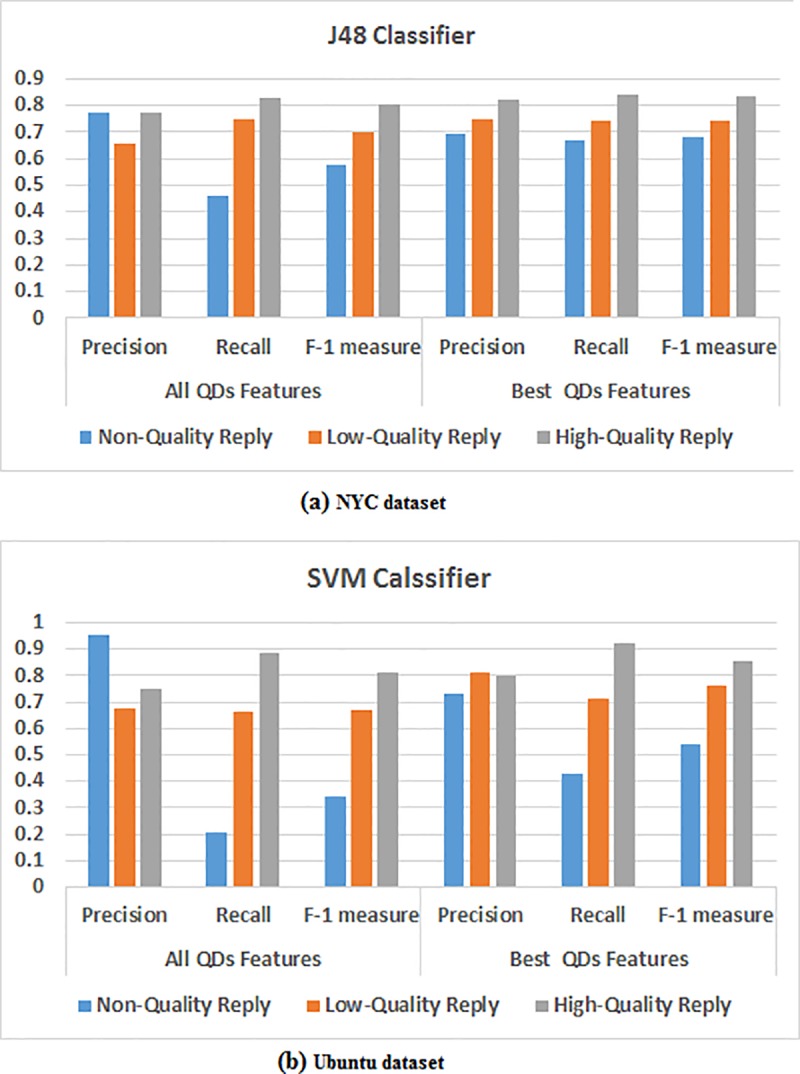
(a) and (b) A comparative analysis between all quality features and best quality features for every class using the best classifier for two datasets.

Fig 5A and 5B present the classification results for the all QDs features and the best QDs features, and a summary of the results for the three classes using the best classifier for the NYC and the Ubuntu datasets, respectively, based on their precision, recall and F-1 measure. It was observed that for the two datasets, the majority of the replies were classified in the high-quality class rather than in the other classes. Also, a higher number of replies were classified in the low-quality class compared to the non-quality class. High values for the F-1 measure in the high-quality class indicated that the replies in this class contained the most significant information that was relevant to the Initial-Post. On comparing, results obtained via best QDs features were clearly seen to be better versus the results obtained via all QDs features by employing three measures. To conclude, the best results were obtained when the classification algorithms were combined with the features selection techniques.

### 7.3 Measuring performance

In this section, assessing of the measures for classification algorithms with the features selection techniques in terms of performance measures was done.

#### 7.3.1 Confusion matrix

For each dataset, evaluation of the confusion matrix was done for best QDs features via the best classifier. Firstly, a confusion matrix can be defined as a specific table layout that enables visualising the algorithms’ performance. In this, the instances in an identified class are signified by each column of the matrix), while instances in an actual class are denoted by each row (or vice versa) [91]. The results pertaining to algorithm testing can be summarised through a confusion matrix for additional inspection as presented in Tables 9 and 10. In both tables, every class was represented by high-quality, low-quality and non-quality replies. The diagonal of the tables (highlighted in bold) contains all correct identifications, making visual inspection of the tables for identifying errors much easier, as these are denoted by the values outside the diagonal.

**Table 9:** Confusion matrix for the best classifier for the NYC dataset.

NYC Dataset (J48 Classifier)		Predicted		
		High-Quality	Low-Quality	Non-Quality
**Model**	**High-Quality**	**288**	41	13
	**Low-Quality**	41	**225**	37
	**Non-Quality**	21	36	**114**

**Table 10:** Confusion matrix for the best classifier for the Ubuntu dataset.

Ubuntu Dataset (SVM Classifier)		Predicted		
		High-Quality	Low-Quality	Non-Quality
**Model**	**High-Quality**	**410**	28	6
	**Low-Quality**	58	**166**	9
	**Non-Quality**	45	10	**41**

The actual classes for the two datasets were defined in Section 6 in Table 5. The confusion matrix for the NYC dataset shown in Table 9 is discussed as follows: First of all, the model demonstrated that for the 342 actual high-quality classes, the majority of the replies are 288 actually belonging to the high-quality class, and were correctly classified (84.21%). Based on the model, 41 replies were inaccurately identified as low-quality (11.99%) and 13 replies (3.80%) were inaccurately classified as those belonging to the non-quality class, even though they were able to provide information that was totally relevant to the final user. Second, for 303 actual low-quality class, the model depicted the majority of the replies were 225 actually belonging to the low-quality class, and were correctly classified (74.26%). However, 41 actually belonged to low-quality replies and were inaccurately classified as those belonging to the high-quality class (13.53%), while 37 belonging to low-quality replies were inaccurately classified as non-quality replies (12.28%). These results showed that although the replies possessed characteristics similar to the high-quality and non-quality class, respectively, they could only provide information that was partially relevant to the final user. Lastly, for 171 actual non-quality class, the model indicated that most replies 114 actually belonging to the non-quality class were correctly classified (66.67%), 21 replies (12.28%) of the non-quality replies were incorrectly classified as high-quality replies, while 36 replies (21.05%) were incorrectly classified as low-quality replies. These results showed that although the replies had some characteristics that were similar to the low-quality rather than high-quality replies, they provided no relevant information to the final user. Based on the confusion matrix pertaining to the diagonal values, the model was seen to be capable in identifying the three classes well.

Similarly, in the case of the Ubuntu dataset in Table 10, the model was as follows: First, for the 444 actual high-quality class, the model showed that the majority of replies, 410 which actually belonged to the high-quality replies class were correctly classified as being of high-quality (92.34%) as they could provide information that was totally relevant to the final user. Meanwhile, 28 replies (6.31%) and 6 replies (1.35%) were inaccurately classified as those belonging to the low-quality and non-quality class, respectively, even though they could provide information that was totally relevant to the final user. They possessed characteristics that were similar to the low-quality and non-quality classes, respectively. Second, for 233 actual low-quality class, the model indicated the majority of replies 166 actually belonged to low quality class, and were appropriately classified (71.25%) as being of low-quality, while 58 actual replies (24.89%) of the low-quality were incorrectly classified as high-quality, while 9 actual replies (3.86%) of the low-quality replies were incorrectly classified as non-quality replies. These results showed that although the replies possessed characteristics that were similar to the high-quality instead of the non-quality replies, they could only provide information that was partially relevant to the final user. Lastly, the model demonstrated that for the 96 actual non-quality class, the 41 replies (42.7%) were classified correctly as non-quality since they offered irrelevant information for the final user. Meanwhile, the classifier incorrectly classified 45 replies (46.88%) of the non-quality replies as high-quality ones. Based on the confusion matrix, the authors could observe that the model in question faced difficulty in differentiating low-quality replies as being high-quality replies. Ten replies (10.42%) of the non-quality replies were inaccurately classified as those belonging to the low-quality class. This was due to the fact that these replies possessed similarities to the high-quality replies rather than to the non-quality replies, and were erroneously considered as being relevant to the Initial-Post by the classifier. Subsequently, the model was found to be better in distinguishing high-quality and low-quality replies versus non-quality replies.

#### 7.3.2 Comparison with related models

A comparison was made by the authors between two baseline and the work provided by the authors [17] to evaluate the performance of the put forward model. The two datasets were employed to assess the three models. A comparison was done for these and the put forward classified quality Initial-Post replies pairs. Frist, in the case of the rule-based model, it was assumed by the authors that the Initial-Post in a thread is considered as a question while all replies in the same thread were branded as solutions. Second, in the BoW model, lexical characteristics pertaining to the text between IPR pairs that had to be classified were captured by the authors. For the purpose of classification, frequency of words in IPR pairs was employed as features by the authors. For a range of text classification works, BoW-based classifiers are commonly employed. Last comes the role pertaining to the user posts model. The authors employed a range of features such as structural features, content-based features, sentiment-based features and user features to segment initial post replies pairs into eight classes that follow initial post as question and replies in the form of repeat question, clarification, further details, solution, positive feedback, negative feedback, and junk.

For each dataset, the results pertaining to all QDs features as well as the best QDs features with the baselines were compared by the authors. Table 11 presents the classification results for the IPR pairs carried out with the help of the quality model classifiers and the baseline classifiers (Rule based, BoW) as well as the Role of user posts [17]. In terms of best QDs features pertaining to the NYC dataset, the results showed an overall classification accuracy of 76.83%, along with the value of precision (0.767), recall (0.768) and F1-measure (0.768). Likewise, in the Ubuntu dataset, the result showed a classification accuracy of 79.82%, along with the values of precision (0.795), recall (0.798) and F1-measure (0.788). For the three measures, the results pertaining to all QDs features were listed in Tables 6 and 7.

**Table 11:** Classification results for the rule-based, bag-of-words, role of individual user messages, all quality features and the proposed classification approaches.

**NYC Dataset**					
**Metrics**	**Rule based**	**Bag of words (BoW)**	**Role of user posts**	**All QDs features**	**Best QDs features** **(Proposed approach)**
**Accuracy**	61.88%	60.98%	75.11%	72.30%	**76.83%**
**Precision**	0.441	0.596	0.726	0.730	0.767
**F1-measue**	0.499	0.529	0.724	0.716	0.768
**Ubuntu Dataset**					
**Metrics**	**Rule based**	**Bag of words (BoW)**	**Role of user posts**	**All QDs features**	**Best QDs features** **(Proposed approach)**
**Accuracy**	58.03%	57.66%	72.69%	73.48%	**79.82%**
**Precision**	0.442	0.503	0.705	0.754	0.795
**F1-measue**	0.471	0.473	0.712	0.712	0.788

Thus, the use of the suggested quality features set yielded different results for the two datasets due to the nature of each dataset. Note that the NYC dataset is a general discussion forum while the Ubuntu dataset is a specific domain discussion forum. Furthermore, the proposed classifier was able to significantly outperform the baseline classifiers for the three metrics that were studied. Here the Initial-Posts were considered as a query and the replies as the documents for determining the relevant (high-quality), less relevant (low-quality), and irrelevant (non-quality) replies to the Initial-Post in order to identify the relevant replies (high-quality) of the IPR pairs. On the other hand, based on the results mentioned in Tables 6, 7, 8 and 11, that the proposed quality dimensions features displayed differing performances based on the forum types and contents. The QDs could show different selecting effects for different algorithms and two forums domains. Therefore, based on our finding, it is found that the J48 classifier showed the best result in NYC dataset. However, the SVM classifier showed the best result in the Ubuntu dataset, while the NB classifier showed the least result in the two datasets (NYC and Ubuntu). As discussed previously, assessment of feature selection techniques was done; to achieve the best QDs features, analysis of all QDs features was done. Thus, the best result was obtained when the classification algorithm was combined with the features selection techniques. It was important to recognise such accurate replies in order to successfully apply in text forum thread summarization, question-answer pair detection, forum search, etc.

## 8.0 Conclusions and future work

In this study, human judgment and the quality dimensions features for identifying the best quality features were exploited to detect the relevant user replies to the Initial-Posts in a discussion thread (IPR pairs) to help in detecting the quality of the user replies in the TFThs. Six QDs features were studied using the discussion thread structure for assessing the user reply quality, which included the relevancy, author activeness, timeliness, ease-of-understanding, amount-of-data, and politeness dimension features. Thereafter, the values of the quality features for every reply were estimated. Human judgment was also used to classify the replies as high-quality, low-quality or non-quality. The SVM, NB and J48 classifiers were applied to classify the replies in any one out of the three groups mentioned above. Additionally, the features selection techniques of Information Gain, Chi-square and Gain Ratio were used as these were better indicators for identifying the quality of the replies along with the best quality dimensions features. According to these experiments, the model was able to identify the appropriate quality features from the six QDs features for the TFThs, thereby improving the extraction of high-quality replies from the thread. Furthermore, this model also possessed a good classification ability which helped in identifying the high-quality users. It is believed that this proposed model will be able to support content filtering and specific forum searches. In future, this work can be further expanded to include text forum threads summarization.
